# Genetic variation at MHC class II loci influences both olfactory signals and scent discrimination in ring-tailed lemurs

**DOI:** 10.1186/s12862-019-1486-0

**Published:** 2019-08-22

**Authors:** Kathleen E. Grogan, Rachel L. Harris, Marylène Boulet, Christine M. Drea

**Affiliations:** 1 University Program in Ecology, Duke University, Durham, NC USA; 2 Department of Evolutionary Anthropology, Duke University, Durham, NC USA; 3 Department of Psychology, Emory University, Atlanta, GA USA; 4 Department of Biological Sciences, Bishop’s University, Sherbrooke, Canada; 5 Department of Biology, Duke University, Durham, USA; 6 Pennsylvania State University, 516 Carpenter Building, University Park, PA 16802 USA

**Keywords:** MHC-DRB, Genetic diversity, Strepsirrhine primate, Chemical signal, Scent mark, Sexual selection

## Abstract

**Background:**

Diversity at the Major Histocompatibility Complex (MHC) is critical to health and fitness, such that MHC genotype may predict an individual’s quality or compatibility as a competitor, ally, or mate. Moreover, because MHC products can influence the components of bodily secretions, an individual’s body odors may signal its MHC composition and influence partner identification or mate choice. Here, we investigated MHC-based signaling and recipient sensitivity by testing for odor-gene covariance and behavioral discrimination of MHC diversity and pairwise dissimilarity in a strepsirrhine primate, the ring-tailed lemur (*Lemur catta*).

**Methods:**

First, we coupled genotyping of the MHC class II gene, DRB, with gas chromatography-mass spectrometry of genital gland secretions to investigate if functional genetic diversity is signaled by the chemical diversity of lemur scent secretions. We also assessed if the chemical similarity between individuals correlated with their MHC-DRB similarity. Next, we assessed if lemurs discriminated this chemically encoded, genetic information in opposite-sex conspecifics.

**Results:**

We found that both sexes signaled overall MHC-DRB diversity and pairwise MHC-DRB similarity via genital secretions, but in a sex- and season-dependent manner. Additionally, the sexes discriminated absolute and relative MHC-DRB diversity in the genital odors of opposite-sex conspecifics, suggesting that lemur genital odors function to advertise genetic quality.

**Conclusions:**

In summary, genital odors of ring-tailed lemurs provide honest information about an individual’s absolute and relative MHC quality. Complementing evidence in humans and Old World monkeys, we suggest that reliance on scent signals to communicate MHC quality may be important across the primate lineage.

**Electronic supplementary material:**

The online version of this article (10.1186/s12862-019-1486-0) contains supplementary material, which is available to authorized users.

## Background

The Major Histocompatibility Complex (MHC) is an extremely polymorphic group of genes within the adaptive immune system of vertebrates that plays a critical role in disease resistance [93]. Because genetic diversity at the MHC is fundamentally linked to parasite resistance, survivorship, and reproductive success [93, 113], an individual’s MHC genotype is hypothesized to be an important predictor of its quality as a mate. If MHC-based information is recognizable to others, animals could increase their reproductive success by selecting mates that possess particular MHC genotypes, such as diverse alleles or specific alleles which convey disease resistance [74, 91]. Although researchers have found evidence that MHC genotype influences mate choice or its proxies in many species (reviewed in [58]), the mechanism by which animals assess the MHC of conspecifics is still under investigation (reviewed in [98]). Given that the protein products of the MHC can influence body odor, scientists have implicated an olfactory-based mechanism (reviewed in [8, 126]); however, researchers rarely combine chemical and behavioral approaches within the same study to test the purported mechanism of information transfer [70, 79]. Here, using the ring-tailed lemur (*Lemur catta*) – a strepsirrhine primate for which there is strong evidence of condition-dependent olfactory signaling [10, 16, 23, 44] – we test for both olfactory-based MHC advertisement and recognition.

Because MHC diversity is critical to an individual’s current health and the health of its offspring, potential mates or social partners might be chosen for their MHC diversity (i.e., quality), for their possession of a particular disease-resistant allele or for their MHC dissimilarity relative to the chooser [78, 113]. For mating and social behavior to be influenced by the MHC, however, individuals must both indicate their respective MHC genotype and be able to evaluate the MHC information in the signals of conspecifics [8, 47]. Previously, researchers have shown that condition-dependent signals of quality can be used by both sexes to assess potential partners [18, 54, 89, 90]. Although evidence of correlation with MHC genotype has derived primarily from visual signals, such as antler size [25] or bright coloration [107], chemical signals could prove more reliable for advertising MHC genotype [7, 74, 88, 126]: Notably, because degraded MHC molecules are shed from the cell surface and found in body fluids (e.g. serum, saliva, sweat, urine, and glandular secretions), they may function directly as olfactory cues [8, 79, 111]. MHC molecules may also bind relevant volatile compounds, forming a ligand-MHC molecule complex that may stimulate the olfactory senses ([3, 70, 122], but see [62]). Lastly, the MHC may influence the composition of the host’s microbiota [5, 64, 127], including those dwelling within scent glands that contribute to volatile chemical production [37, 65, 67, 118]. Among taxa that display MHC-associated mate choice, researchers have implicated the operation of an olfactory mechanism in fish [1, 79, 95], reptiles [82], birds [31, 66, 68], and mammals [94, 124], including humans (reviewed in [45, 123]).

The ring-tailed lemur is a fitting model for an odor-based test of MHC advertisement (e.g. [61]) and discrimination of conspecific quality. Endemic to Madagascar, ring-tailed lemurs are an endangered species [2] that, owing to population decline and habitat fragmentation [22, 101], faces the threat of inbreeding and inbreeding depression, whether in the wild or in captivity [17, 42, 85]. They live in female-dominated, multi-male, multi-female societies characterized by strictly seasonal breeding and an elaborate system of olfactory reproductive advertisement [55]. Beyond the male’s specialized antebrachial and brachial scent glands [80, 102] and associated wrist-marking behavior [59], both sexes possess genital scent glands, the secretions of which are unusually chemically complex [106]. We focus on these labial and scrotal secretions because both sexes deposit these secretions through genital marking and these labial and scrotal secretions share ~ 170 volatile compounds [9]. The diversity and relative abundance of these chemicals in these genital secretions contain information about the signaler’s sex, breeding condition, injury status, individual identity, and genome-wide microsatellite diversity (or neutral heterozygosity), as well as its relatedness to other individuals [9, 10, 16, 23, 24, 44, 106]. Moreover, this chemically encoded information is salient and distinguishable to conspecifics [18, 23, 44, 105]. Thus, lemur genital odors honestly advertise at least one measure of genetic quality and relatedness in both sexes.

We combined MHC genotyping with chemical analyses of genital secretions and behavioral tests of scent discrimination to ask 1) if lemurs also advertise their MHC-DRB quality and dissimilarity via chemical cues and 2) if opposite-sex conspecifics can detect this olfactory information. We genotyped captive ring-tailed lemurs (*N* = 62) at the most diverse class II MHC gene, DRB [41], and analyzed the volatile chemical composition of their genital secretions. We used next generation sequencing to genotype the MHC-DRB gene [41], investigating both allelic MHC-DRB diversity and functional MHC-DRB diversity by collapsing alleles into ‘supertypes,’ or groups of alleles with similar immunogenetic binding properties despite different nucleotide sequences [42]. Using gas chromatography and mass spectrometry, we analyzed both the overall, volatile chemical composition of genital secretions, as well as a subset of compounds, including fatty acids (FAs) and fatty acid esters (FAEs), identified a priori based on their putative linkage to fertility in some female primates [26, 73, 77] and their relation to microsatellite diversity in female ring-tailed lemurs [10]. Lastly, we used behavioral testing to determine if conspecifics can discriminate between absolute diversity and relative dissimilarity in MHC-DRB genotypes based on scent alone.

## Results

### Signaling of individual MHC quality via odor-gene covariance

**Fig. 1:**
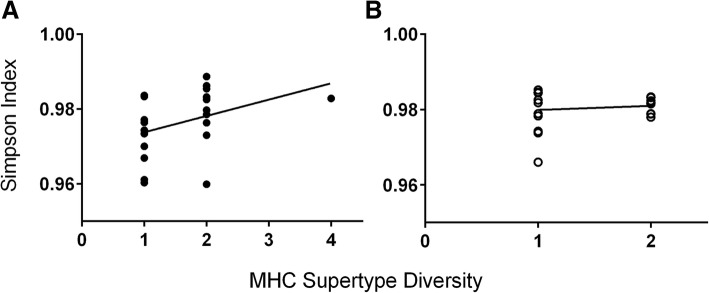
Linear regression (black line) showing the seasonal relationships between chemical diversity of all compounds in scrotal secretions and MHC-DRB supertype diversity in male ring-tailed lemurs in the **a** breeding season (closed circles) and **b** nonbreeding season (open circles)

**Fig. 2:**
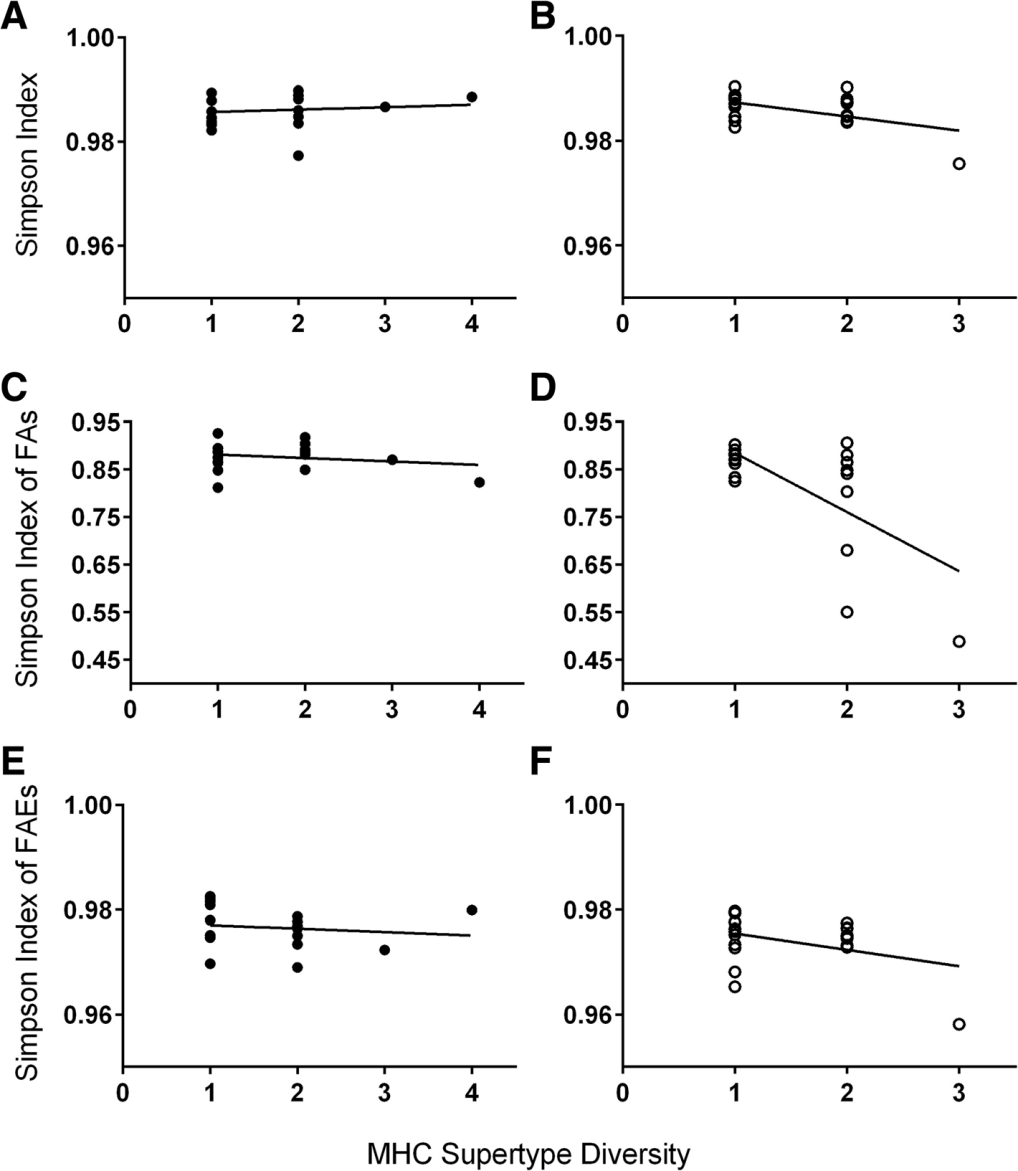
Linear regression (black line) showing seasonal relationships between different measures of chemical diversity (Simpson index of all compounds: **a**, **b**; Simpson index of FAs: **c**, **d**; Simpson index of FAEs: **e**, **f**) in labial secretions and MHC-DRB supertype diversity in female ring-tailed lemurs in the **a**, **c**, **e** breeding season (closed circles) and **b**, **d**, **f** nonbreeding season (open circles)

We found that both sexes of ring-tailed lemurs signaled their individual MHC-DRB quality via the chemical compounds expressed in their genital secretions. Male MHC-DRB diversity was significantly and positively correlated with chemical diversity (*N* = 23, Z = 2.17, *P* = 0.03), regardless of season (Fig. 1; Table 1; Additional file 1: Tables S3A and S4). By contrast, female MHC-DRB diversity was unrelated to overall chemical diversity in either season (*N* = 20, Z = 0.24, *P* = 0.81; Fig. 2; Table 1; Additional file 1: Table S3B). Nevertheless, female MHC-DRB diversity was significantly and negatively correlated to the diversity of an important subset of chemicals, FAs, but only during the nonbreeding season (Z = − 3.75, *P* = 0.001; Fig. 2; Table 1; Additional file 1: Table S5A). Female MHC-DRB diversity was not related to the diversity of FAEs, regardless of season (Z = − 0.34, *P* = 0.740; Fig. 2; Table 1; Additional file 1: Table S5B). For both sexes, removal of the most MHC-diverse individual did not alter the results (Additional file 1: Tables S3, S4 and S5).

**Table 1:** Relationships between the Simpson index of chemical diversity and MHC-DRB diversity in ring-tailed lemurs across seasons, with significant relationships indicated in bold

Sex	Compounds in Simpson index	Explanatory variable	Z value	*P* value	Effect
Male	Overall Diversity	Season	1.64	0.10	Simpson diversity index increases with increasing MHC diversity, regardless of the season
		MHC_supertype_	**2.17**	**0.03**	
		Season x MHC_supertype_	− 0.89	0.37	
Female	Overall Diversity	Season	0.86	0.390	No relationship between Simpson index and season or MHC diversity in females
		MHC_supertype_	0.24	0.810	
		Season x MHC_supertype_	−0.98	0.330	
Female	Fatty Acid Diversity	**Season**	**2.24**	**0.025**	Simpson index for FAs is negatively correlated with MHC diversity, but only in the nonbreeding season
		MHC_supertype_	−0.38	0.703	
		Season x MHC_supertype_	**−3.75**	0.001	
Female	Fatty Acid Ester Diversity	Season	1.02	0.310	No relationship between female Simpson index of FAEs and season or MHC diversity in females
		MHC_allele_	−0.34	0.740	
		Season x MHC_allele_	−1.64	0.100

### Signaling of relatedness via dyadic, odor-gene covariance

**Fig. 3:**
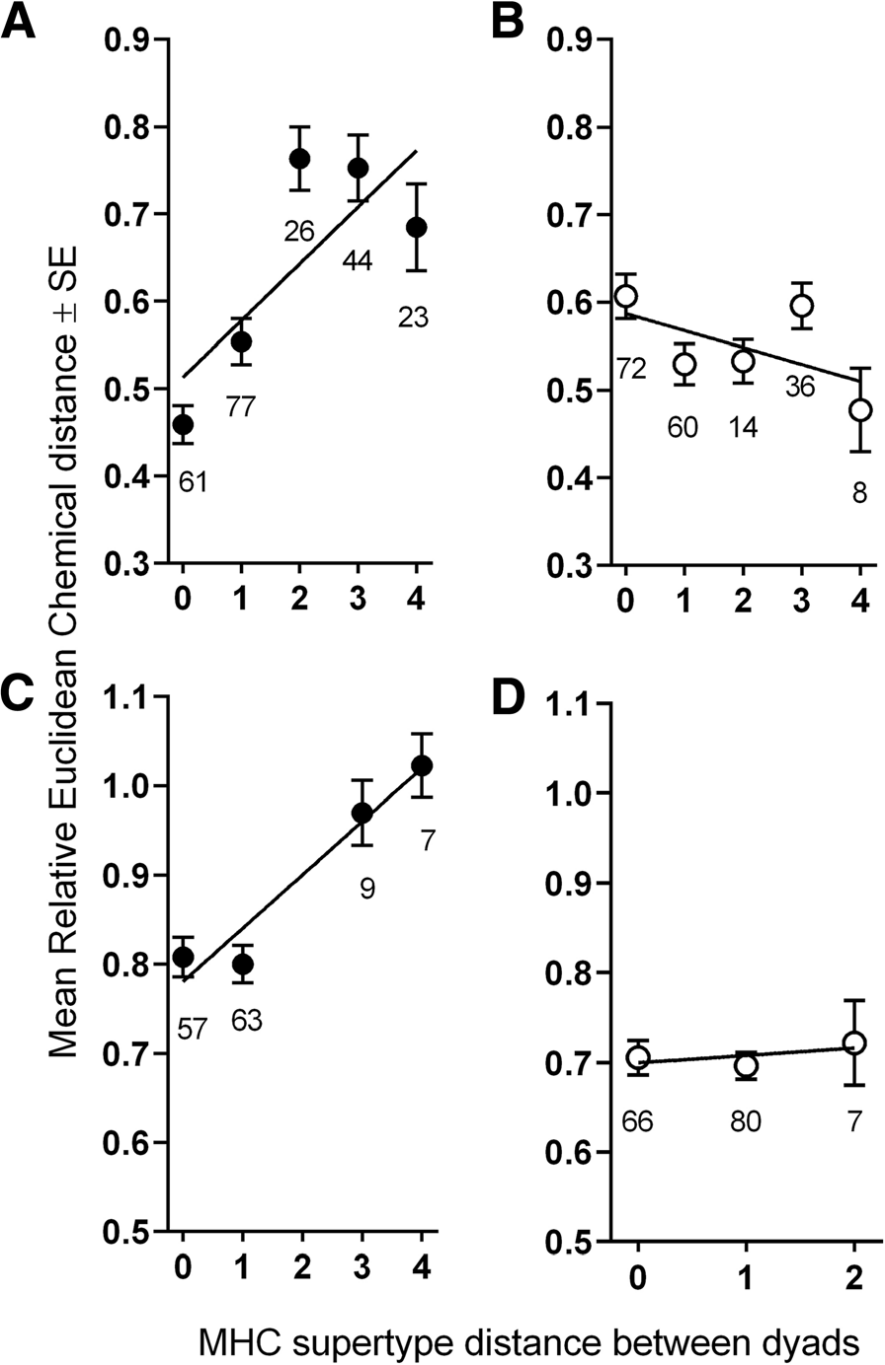
Linear relationships (black lines) between the chemical distance (relative Euclidean) and the genetic distance (number of unique MHC-DRB supertypes, i.e., MHC_supertype diff_) between **a**-**b** male-male and **c**-**d** female-female ring-tailed lemur dyads during the breeding season (closed circles; **a**, **c**) and the nonbreeding seasons (open circles; **b**, **d**). The numbers of dyads are provided below each data point

In all same-sex lemur dyads, genital olfactory cues encoded information about MHC-DRB distance, but in a season-dependent fashion (Fig. 3; Table 2; Additional file 1: Table S6). After controlling for covariates, chemical distances between MM dyads positively correlated with unique MHC-DRB supertypes during the breeding season (*N* = 22 males as 231 MM dyads, *r* = 0.408, *P* < 0.001, Fig. 3a), but not during the nonbreeding season (*N* = 20 males as 190 MM dyads, *r* = − 0.079, *P* = 0.270, Fig. 3b). Similarly, for FF dyads, we observed a significant, positive correlation during the breeding season between the number of unique MHC-DRB supertypes and chemical distance (*N* = 17 females as 136 FF dyads, *r* = 0.313, *P* < 0.001, Fig. 3c), that was not apparent during the nonbreeding season (*N* = 18 females as 153 FF dyads, *r* = 0.027, *P* = 0.729, Fig. 3d).

**Table 2:** Partial Mantel tests showing the seasonal relationships between the relative Euclidian chemical distance (for genital odorants) and MHC-based genetic distance for same-sex (MM and FF) dyads of ring-tailed lemurs

Dyad type	Variable	Number of unique MHC-DRB supertypes					
		Breeding season			Nonbreeding season		
		SS	*r*	*P*	SS	*r*	*P*
MM dyads	MHC	**2.309**	**0.408**	**< 0.001**	0.042	−0.079	0.276
	Age	0.203	0.121	0.068	**0.139**	**0.145**	**0.005**
	Housing	0.157	0.106	0.101	0.006	−0.029	0.692
	Month of collection	0.006	−0.020	0.763	*0.124*	*0.137*	*0.062*
FF dyads	MHC	**0.391**	**0.313**	**< 0.001**	0.003	0.027	0.738
	Age	0.001	0.014	0.872	0.008	−0.047	0.560
	Housing	0.029	0.085	0.323	0.004	0.034	0.679
	Month of collection	*0.093*	*0.153*	*0.076*	**1.461**	**0.623**	**< 0.001**

Chemical distance is based on 203 and 338 compounds for MM and FF dyads, respectively. Tests include three socio-demographic and environmental variables as covariates. Sums of squares (SS) and partial Mantel correlation coefficients (*r*) with significant *P* values (*P* ≤ 0.05) are shown in bold type, whereas trending values (*P* ≤ 0.10) are shown in italics

**Fig. 4:**
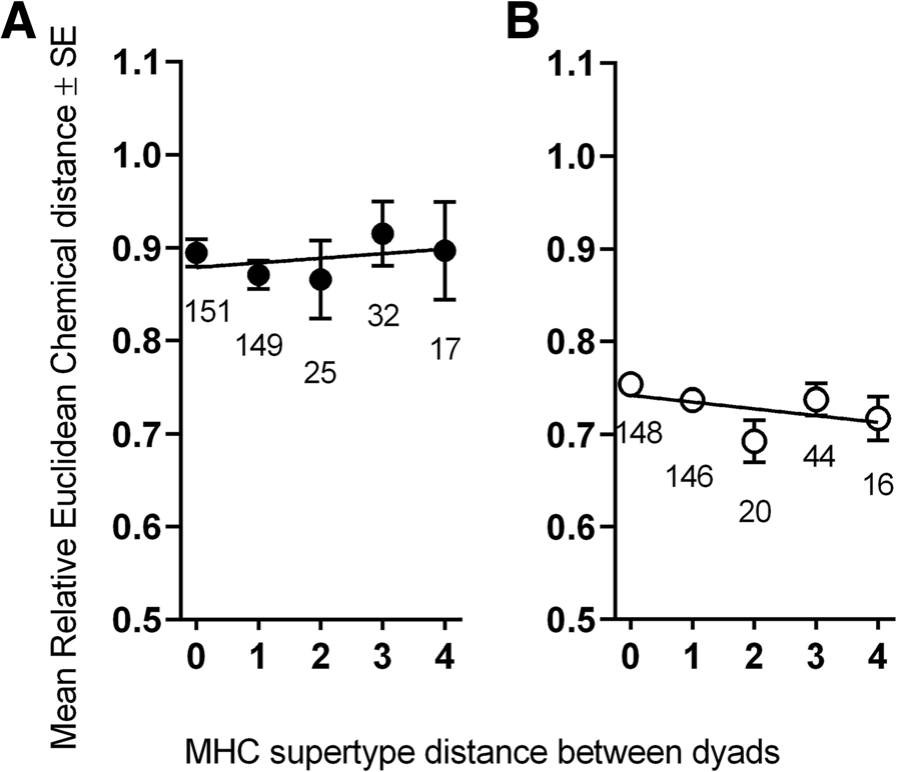
Linear relationships, indicated by black line, between the chemical distance (relative Euclidean) and the number of unique MHC-DRB supertypes (i.e., MHC_supertype diff_) for male-female dyads of ring-tailed lemurs during the **a** breeding season (closed circles) and **b**) nonbreeding season (open circles). The numbers of dyads are provided below each data point

We could not detect any relationship between chemical distance and MHC distance between MF dyads in the breeding season (*N* = 39 subjects of both sexes combined as 374 MF dyads, *r* = 0.0014, *P* = 0.8280; Fig. 4a), but there was a trending negative relationship between mixed-sex dyads during the nonbreeding season (*N* = 38 subjects of both sexes combined as 360 MF dyads*, r* = − 0.0099, *P* = 0.0647; Fig. 4b).

### Olfactory discrimination of MHC genotype between mixed-sex conspecifics

**Fig. 5:**
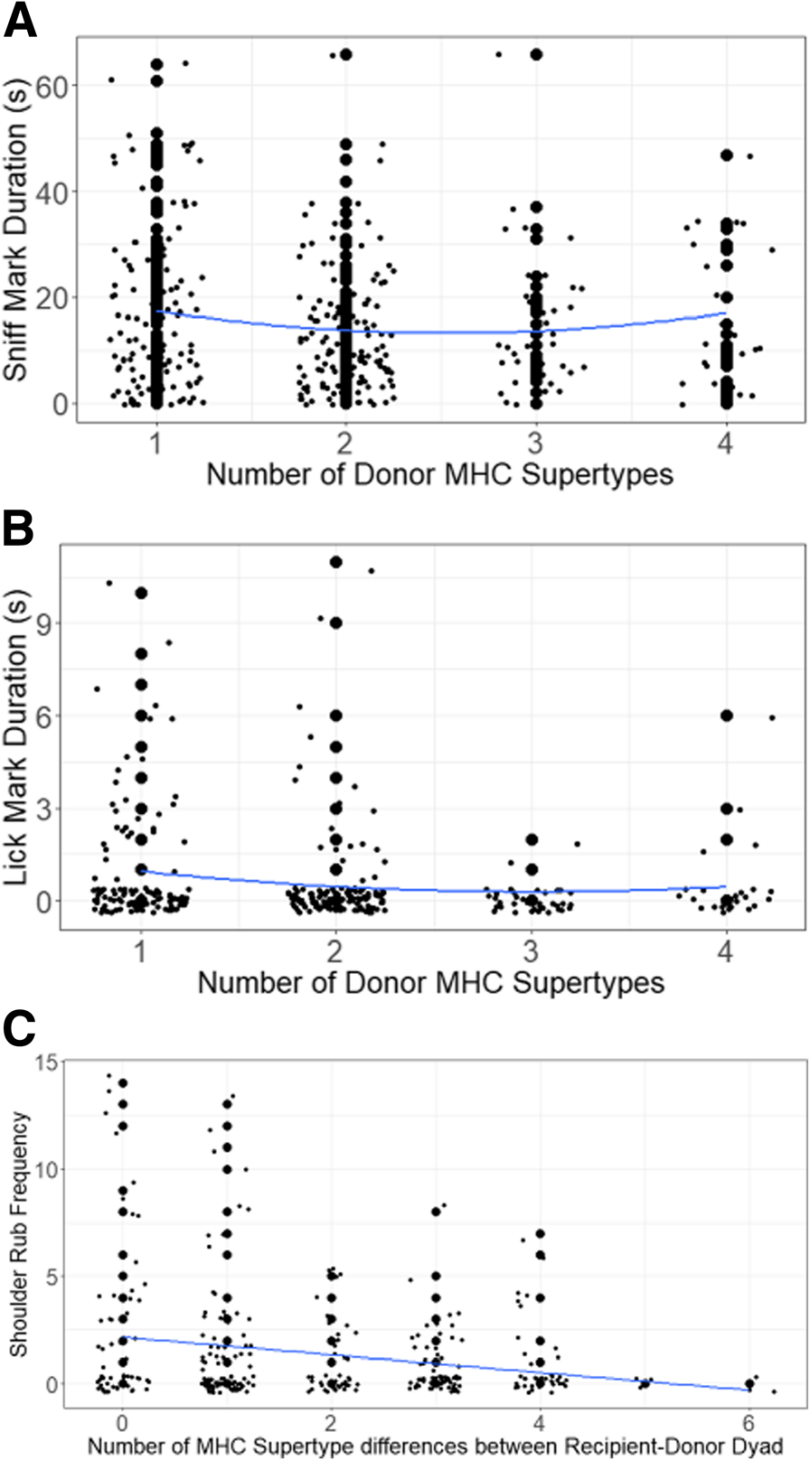
Behavioral response (**a**: sniff mark duration; **b**: lick mark duration; **c**: shoulder rub frequency) of male ring-tailed lemurs to the odorants of female conspecifics. The line shows the regression and points are jittered to avoid overlap of the data

**Fig. 6:**
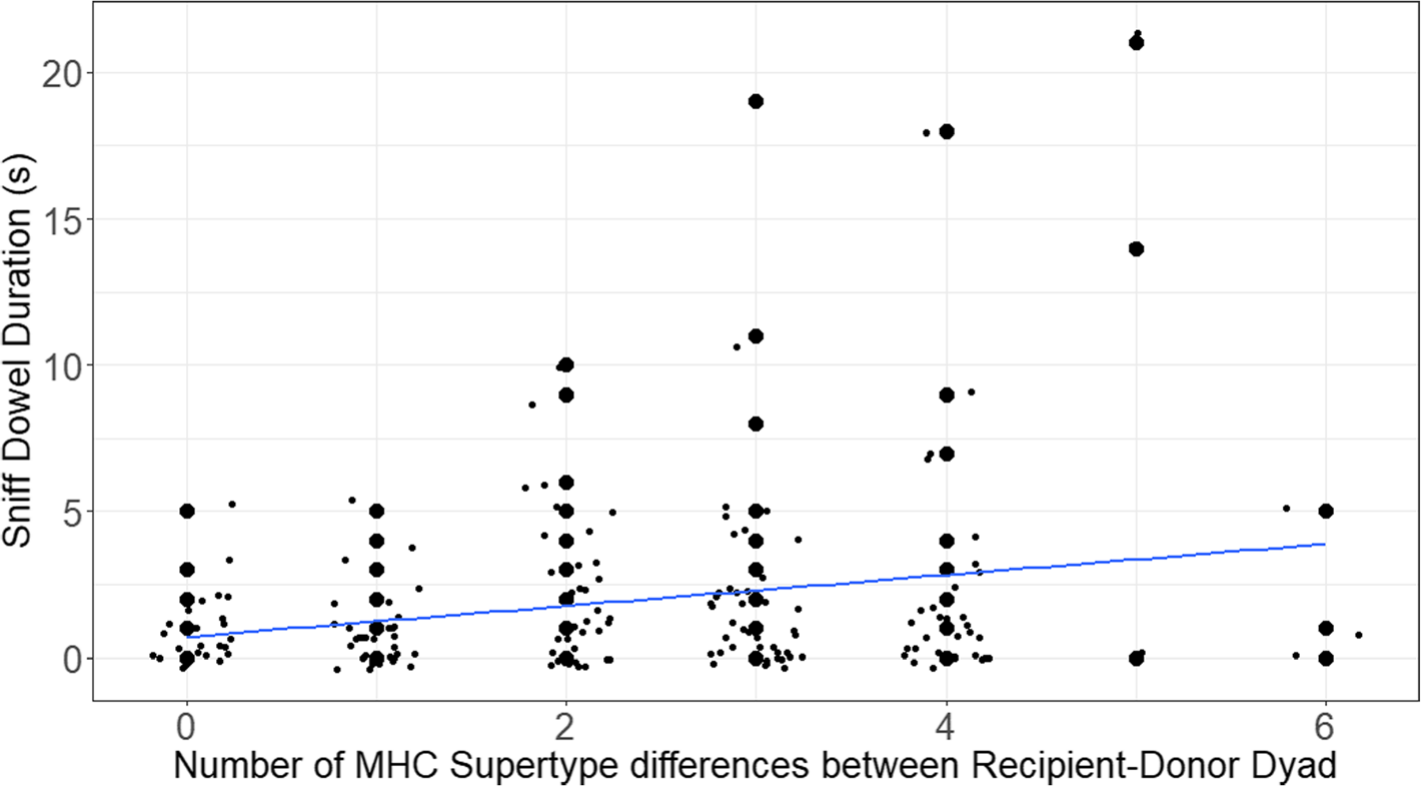
Behavioral response of female ring-tailed lemurs, i.e., time spent sniffing the area on the dowel adjacent to the odorant of the male conspecifics. The line shows the regression and points are jittered to avoid overlap of the data

Although we could only detect the chemical signaling of MHC-DRB diversity in males in the breeding season, behaviorally both male (Table 3; Fig. 5) and female (Fig. 6) recipients showed significant discrimination between the genital secretions of opposite-sex, conspecific donors based on their possession of different MHC-DRB genotypes. The pattern of response to conspecific secretions, however, differed between the sexes.

**Table 3:** Relationship between the MHC-DRB genotype of female odorant donors and the behavior that male recipients directed toward the female’s scent mark, with significant relationships indicated in bold. Explanatory variables with superscripts indicate the quadratic variable, whereas those without superscripts are linear

Behavior	Best-fit explanatory variable	slope	Z value	*P* value	Effect
Proximity	MHC_supertype diff_	−0.08	−1.5	0.13	No relationship between the male’s time in proximity and the female’s MHC dissimilarity
Sniff mark	MHC_donor_	**−0.55**	**−2.46**	**0.014**	More time spent by males sniffing the marks of female donors with intermediate supertype diversity
	MHC_donor_^2^	**0.10**	**2.13**	**0.033**	
Lick mark	MHC_donor_	**−4.24**	**−4.36**	**< 0.001**	Longer time spent by males licking the marks of female donors with intermediate MHC diversity
	MHC_donor_^2^	**0.82**	**4.01**	**< 0.001**	
Sniff dowel	MHC_supertype diff_	0.17	1.16	0.250	Longer time spent by males sniffing the area adjacent to the marks of females when the supertype differences were intermediate between dyads
	MHC_supertype diff_^2^	**−0.06**	**−1.96**	**0.050**	
Shoulder rub	MHC_supertype diff_	**−0.21**	**−2.1**	**0.035**	Fewer shoulder rubs by males with increasing supertype differences between the recipient-donor dyad

Male recipients (*N* = 18) investigated female secretions more (i.e., spent more time sniffing and licking them) if the donors (*N* = 29) were of intermediate diversity at the MHC-DRB than if they were at either end of the MHC-DRB diversity spectrum (Table 3; Fig. 5). Additionally, as the relative MHC-DRB dissimilarity of female donors increased, male recipients performed fewer shoulder rubs (Table 3).

Female recipients (*N* = 9) did not investigate (sniff: Z = − 0.86, *P* = 0.39; lick: Z = − 1.2, *P* = 0.23) male scent ‘marks’ according to the MHC-DRB diversity of the donors (*N* = 17); nevertheless, they differentiated their responses towards the areas adjacent to the male’s mark. Specifically, as the MHC-DRB supertype dissimilarity of the male donor increased, female recipients spent more time sniffing areas adjacent to the mark (slope = 0.226, Z = 2.37, *P* = 0.018; Fig. 6).

## Discussion

Owing to its role in survival and reproductive success, immunogenetic diversity is an important predictor of individual quality and may be signaled via visual or chemical means. Our study provides support for the socially salient, chemical signaling of genetic quality in a strepsirrhine primate. Despite sex differences in the chemical ‘indicators’ of quality and their seasonal emergence, ring-tailed lemurs of both sexes signaled their MHC-DRB diversity and dissimilarity to conspecifics via the volatile component of their genital secretions. Moreover, both sexes were able to use these and potentially other olfactory cues to discriminate relevant information about the MHC genotypes of opposite-sex conspecifics. These results confirm the functional significance of our previous work showing detectable relationships between chemical diversity and microsatellite diversity in both sexes [10, 16, 18]. Our results also provide a foundation from which to explore if, using data on reproductive success from wild populations, ring-tailed lemurs actually choose mates according to diversity or dissimilarity of their MHC-DRB genotype.

Male ring-tailed lemurs appear to advertise their genetic quality both via MHC-DRB diversity and via microsatellite diversity. Moreover, they appear to do so in a similar fashion, in that both measures of genetic diversity were positively correlated with the overall chemical diversity of scrotal secretions. Although the relationship with microsatellite diversity only emerged in the breeding season [16], the MHC odor-gene covariance in males emerged regardless of season (albeit more strongly in the breeding season). Female ring-tailed lemurs, on the other hand, signaled their genetic diversity via certain chemicals, specifically FAs. Previously, we had shown that females signaled their increased microsatellite diversity via a negative relationship with the diversity of FAs, a relationship that was only evident during the breeding season [10]. Here, we show that females also advertise MHC-DRB diversity via a negative relationship with FA diversity, but this time the relationship was evident only during the nonbreeding season. It could be that we lacked the power to detect these relationships reliably in both seasons. Alternatively, it could be that contrasting demands in these seasons influence the differential expression of genetic quality in these odor-gene relationships.

In this female-dominant primate, in which female choice is likely to influence male mating success, males may benefit from advertising their genetic quality to females during the breeding season. Males may also benefit from advertising their quality to male competitors year-round. Females, however, may be relaying different information depending on the season. During the breeding season, signaling genome-wide microsatellite diversity and relatedness may be critical to avoid inbreeding [9, 10]. In contrast, signaling MHC-specific diversity and health during the nonbreeding season might convey competitive ability during periods of intense female-female competition (e.g. [44, 71]) and energetically expensive lactation [83]. Additionally, intragroup female competition for access to resources increases during the nonbreeding season [39, 100]. During these social disputes, the killing of vulnerable infants, committed by both sexes, is a significant risk [19, 48, 53, 56, 60]. Signaling one’s health and vitality may reduce the likelihood of aggressive encounters that could lead to infanticide by competing females (reviewed in [116]).

Our results contrast the lack of odor-gene covariance found in mandrills (*Mandrillus sphinx*), the only other primate in which a relationship between chemical secretions and MHC diversity has been investigated. In both male and female mandrills, MHC-DRB diversity was unrelated to the chemical diversity of secretions obtained from the surface of the sternal gland [109]. MHC information, however, may be signaled through other aspects of the animals’ olfactory signatures that were not analyzed by these authors. For instance, just as female ring-tailed lemurs signal MHC-DRB and microsatellite diversity through a subset of chemicals (e.g. FAs; [10]), so too might MHC-DRB information be contained in the ratios or relative abundances of specific odoriferous compounds. Alternatively, socially relevant information may be encoded in the non-volatile portion of secretions [6, 11, 52, 63] or be signaled through the composition of the microbiota present in the scent glands [5, 8, 65, 86, 127] and the odorants they produce [37, 67, 118]. Further exploration of individual compounds, specific subsets of chemicals, or the non-volatile fraction of secretions might yield a signaling pattern that conveys information about MHC genotype. Such evidence would support findings that male mandrills appear to use the MHC genotype of a potential mate for mate-guarding decisions [110] and that MHC diversity is correlated with male reproductive success [108].

The chemical composition of lemur genital secretions also signals MHC-DRB dissimilarity between male-male, female-female, and male-female dyads, echoing previous results demonstrating the same pattern for microsatellite diversity [9, 10, 16]. Signaling relatedness to any potential social ‘partner’ is likely to be relevant throughout the year, to avoid related competitors or to beneficially direct nepotism [16, 18]. Signaling relatedness or compatibility to opposite-sex conspecifics would be particularly important during the breeding season, to avoid inbreeding and maximize offspring diversity [13, 81, 119]. Evidence now exists that odorants signal MHC dissimilarity within same-sex and opposite-sex dyads in two taxa formerly thought to be primarily visually oriented, namely birds (black-legged kittiwake: [66]; song sparrows: [112]) and anthropoid primates (mandrills: [109]), suggesting greater relevance of olfactory cues than previously suspected.

Regarding behavior, our male recipients responded most to the scent of females that had intermediate MHC-DRB diversity, and they responded least to the odorants of females that were at the extremes of MHC-DRB diversity. It may be that increased investigation reflects a preference, whereas decreased investigation reflects an aversion. For example, the reduced responsiveness of males could indicate avoidance of extreme inbreeding and outbreeding depression [33, 34, 114]. Increased male investigation could reflect that more processing time was required to decipher the female’s potential as a mate, reflecting a trade-off between speed and discrimination accuracy (reviewed in [21]). For example, rats increase the accuracy of their ability to discriminate between odors the longer they sniff the odor, and, for more difficult discrimination tasks, the rate of increase in accuracy is slower [96]. Accordingly, it may have been more challenging for males to identify the potential quality or compatibility of females that had mid-range MHC-DRB diversity. Previously, in a study of microsatellite diversity, we had found that male ring-tailed lemurs spent more time sniffing the secretions of less-related females [18], a pattern that has since been replicated in chimpanzees [46], and which could be explained as a preference for unrelated females and/or as a greater processing demand. Regardless of the direction of the behavioral responses, both sets of findings indicate that male ring-tailed lemurs are minimally able to discriminate conspecifics according to both overall genetic relatedness and MHC-DRB diversity/dissimilarity. Male choice maybe be important in this species due to several factors, particularly limited availability of fertile females and differences in female quality [84]: In this species, females are strictly seasonal and generally fertile only 1–3 times per year for a period of less than 24 h [32, 121], and often cycle somewhat synchronously with other females in the social group [92](Pereira 1991). Thus, both sexes should be choosy about the competitive effort directed towards their potential partners.

Lastly, our finding that female ring-tailed lemurs spent the most time sniffing the vicinity of secretions from MHC-DRB dissimilar males complements previous work showing that females of other species show greater responsiveness to the scents of more MHC dissimilar males than of more MHC-diverse males (e.g. [1, 14]).

We have confirmed an honest olfactory mechanism of ornamentation and potential mate choice, namely via genital odor-MHC gene covariance and discrimination, in both sexes of ring-tailed lemurs. Olfactory information about immunogenetic quality and similarity may also influence general social behavior, specifically for prioritizing agonistic or nepotistic interactions. Female lemurs are expected to be choosy under the traditional paradigm of sexual selection [120]; however, mate choice may be equally important for male ring-tailed lemurs [84]. Our data extend the potential for olfactory-based MHC discrimination across the primate order and add to a growing body of literature suggesting that choice of social partner or mate may depend on both MHC dissimilarity and diversity [58].

## Methods

### Subjects

Our subjects (*N* = 62) derived from three captive populations of ring-tailed lemurs, located at the Duke Lemur Center (DLC, *N* = 24 males, 24 females) in Durham, NC, USA, the Indianapolis Zoo (*N* = 4 males, 8 females) in Indianapolis, IN, USA, and the Cincinnati Zoo (*N* = 2 females) in Cincinnati, OH, USA. All of the animals were healthy adults that were reproductively intact (i.e., neither gonadectomized nor hormonally contracepted) at the time of the study. They were housed in mixed-sex pairs or groups, with similar living conditions and provisioning routines across all three institutions (for more details about DLC housing, see [106]). Notably, all of the animals at all three facilities were fed Purina Monkey Chow with assorted fruits and vegetables and had free access to water. Animal care met with institutional guidelines and was in accordance with regulations of the United States Department of Agriculture. The research protocols were approved by the Institutional Animal Care and Use Committee of Duke University (protocol numbers A245–03-07 & A143–12-05) and by the research directors of each zoo.

### MHC genotyping

Using DNA extracted from whole blood or tissue, we genotyped all of the subjects at the MHC-DRB loci using parallel tagged next-generation sequencing [41]. Briefly, blood samples were obtained by staff veterinarians from the femoral vessels of gently hand-restrained subjects or tissue samples were acquired banked from deceased subjects. These samples were stored at − 20 °C until processing. DNA was extracted using either DNA miniprep kits (Sigma, St. Louis, MO, USA) or DNeasy® Blood and Tissue kits (Qiagen, Valencia, CA, USA). We amplified a 171-bp fragment, excluding primers, of the 270-bp second exon of the MHC-DRB gene. This fragment is the most frequently genotyped MHC loci in non-model primate species, especially in lemur species for which genomic data to design primers are scarce (e.g. [50, 57, 87, 103, 115]). Because this fragment excludes several variable amino acids within the MHC-DRB gene, the total MHC-DRB variability may be underestimated. Nonetheless, because the genotyped fragment represents the most variable part of exon 2, we can use this 171-bp fragment as a proxy of diversity across the 6 exons of MHC-DRB. In previous work on ring-tailed lemurs, we have shown that diversity at this MHC-DRB fragment is representative of diversity across other class II MHC genes [41].

To generate MHC-DRB genotypes, we sequenced pooled amplicons using parallel tagged sequencing on two platforms: Ion Torrent PGM® 314v2 chips (Life Technologies, Grand Island, NY, USA) and 454 Titanium® 1/8th lanes (Roche, Nutley, NJ, USA). True MHC-DRB alleles were distinguished from artefacts using a published workflow [41]. Each ring-tailed lemur possessed a mean ± S.D. of 2.22 ± 0.92 MHC-DRB alleles (range = 1–4; see Additional file 1: Table S1, adapted from [42]).

Because of the degeneracy of the genetic code and similarity in the physiochemical properties of some amino acids, researchers can quantify both nucleotide sequence diversity and ‘functional’ diversity, the latter reflecting the diversity of pathogen proteins that an individual’s MHC proteins can bind. We thus organized the MHC-DRB alleles (*n* = 20) into MHC-DRB ‘supertypes’ (*n* = 13; [42]). Supertypes are groups of MHC alleles that, despite having different nucleotide sequences, have similar antigen binding properties [27, 103], and, thus, are likely to bind the same subset of pathogen peptides. Owing to the functional overlap in their peptide binding properties, alleles within a supertype are also likely to be subject to identical selection pressures [49].

We classified supertypes using a protocol [27] widely used in primate MHC-DRB supertype classification [49, 103, 107, 108]. We first determined the allelic reading frame by aligning the MHC-DRB sequences with the human HLA-DRB sequence [12] to identify antigen binding sites. Then, we identified any amino acid sites under positive selection using the CODEML analysis in PAML (Version 4.7; [125]). For amino acids identified as being under putative positive selection, we imported their physiochemical properties, including hydrophobicity, steric bulk, polarity, and electronic effects [99], into a matrix in Genesis 1.7.6 [117]. Lastly, using hierarchical clustering via Cosine, Euclidean, and Pearson correlation distance methods, we identified supertypes based on antigen binding similarity. A single supertype was defined as the terminal group with no further branching points. All three distance methods clustered all 64 ring-tailed lemur alleles into 27 identical clusters or supertypes. The range in the number of alleles that were collapsed into each supertype grouping was 1–8, with a mean ± S.D. of 2.01 ± 1.54 alleles.

### Genital secretion sample collection

We obtained genital gland secretions from a subset (*N* = 57) of the subjects, hereafter scent ‘donors’ at two of the facilities (see Additional file 1 for a description of the factors limiting sample collection from all subjects). We collected samples at the DLC over a period of 10 years (2003–2013), including during the breeding and nonbreeding seasons (*N* = 24 males, 24 females). We also collected samples at the Indianapolis Zoo during the breeding season of 2011 (*N* = 1 male, 9 females). No secretions were collected from subjects at the Cincinnati Zoo. Because our subjects were in the Northern Hemisphere, we considered samples collected from November to March to be ‘breeding season’ samples and those collected from May to August to be ‘nonbreeding season’ samples [28, 106].

At the DLC, trained handlers carefully caught and gently restrained the animals, which were awake and habituated to these procedures. At the Indianapolis Zoo, collections occurred during the annual physical examinations, performed by Zoo staff members, while the animals were under anesthesia (see Additional file 1 for a discussion of the null effects of handling method on genital secretions). Following published methods [106], we used cotton swabs and forceps, pre-cleaned with methanol and pentane, to collect triplicate samples of genital secretions, per subject, at each collection. We gently rubbed the cotton swab against the scrotal or labial glandular field for 5–10 s, placed the scented swabs in pre-cleaned chromatography vials, and stored the vials at − 80 °C. We have previously shown that individual-specific scent signatures are stable across both years and storage time [23, 30, 44, 106]. Each odorant sample was used only once, for either chemical analyses or bioassay presentation, based upon the season of collection, the number of odorant samples available per individual, and the number of possible recipients to which the odorant could be presented. To maximize the possible bioassay presentations, we prioritized achieving an appropriate sample size for chemical analyses to detect statistical differences rather than analyzing the chemistry of every individual.

### Gas chromatography mass spectrometry (GCMS) and chemical diversity indices

All of the chemical analyses were performed on a subset of the genital secretions collected from subjects (*N* = 43) at the DLC. We used previously published GCMS methods and resulting chemical data to quantify the volatile chemical composition of these secretions (collected from *N* = 23 males, 20 females; [10, 16, 44, 106]). Briefly, we extracted the volatile components of the secretions into 1.5 ml of methyl-*tert*-butyl ether, concentrated the extraction, and analyzed the components on a Shimadzu GCMS-QP2010 instrument (Shimadzu Scientific Instruments) equipped with a Shimadzu AOC-20 series autosampler. The compounds were detected using the automatic peak detector (SOLUTION WORKSTATION software, Shimadzu Scientific Instruments) and the peaks individually verified via consultation with the National Institute of Standards and Technology library (for further details, see [30]).

For analyses of the chemical data, we discarded compounds that had inconsistent retention times, or that did not comprise at least 0.05% of the overall area of the GCMS chromatogram. The remaining compounds (*n* = 203 compounds in scrotal secretions and *n* = 338 compounds in labial secretions) consisted of fatty acids, fatty acid esters, cholesterol derivatives, alkanes, and other unidentified compounds [10, 16, 44, 106]. To represent the overall chemical composition of lemur genital secretions [16], we used three measures of diversity: richness, the Shannon index, and the Simpson index [69, 75]. Richness reflects the absolute number of compounds present per chromatogram, regardless of relative abundance or rarity. By contrast, the Shannon and Simpson diversity indices reflect the relative abundances in different ways: The Shannon index is primarily influenced by common compounds of intermediate abundance, whereas the Simpson index gives more weight to compounds of the greatest relative abundance [16, 75]. We calculated these diversity indices for each individual’s overall chemical profile.

We also calculated these diversity indices for two subsets of chemicals, fatty acids (FAs) and fatty acid esters (FAEs), which are synthesized from FAs [20, 43]. Because FAs have been linked to fertility in certain female primates ([26, 73, 77], although see [36]), we had selected these types of compounds*,* a priori, for examining odor-gene covariance in previous studies [10]. We have shown that both chemical subsets, FAs and FAEs, are correlated with microsatellite diversity of female ring-tailed lemurs during the breeding season and, thus, might be indicators of individual quality in lemurs [10]. Here, we examined the three diversity indices for these compounds, specifically, in both sexes (*n* = 25 FAs in 203 total compounds in scrotal secretions and *n* = 33 FAs in 338 total compounds in labial secretions; *n* = 87 and 112 FAEs in male and female genital secretions, respectively; [10, 23]).

### Behavioral bioassays

To test if ring-tailed lemurs, hereafter ‘recipients,’ can use the secretions of ‘donors’ to discriminate between the MHC genotypes of opposite-sex conspecifics, we conducted 300 behavioral trials or ‘bioassays’ [16, 40, 105]. We used recipients for whom the odorant donors were ‘unknown,’ defined as never having resided concurrently with the recipient in the same group and/or never having had their secretions presented to the recipient in prior bioassays (see Additional file 1). We thus used recipients (*N* = 27) from the multiple institutions, including at the DLC (*N* = 14 males, 5 females), Cincinnati Zoo (*N* = 2 females), and Indianapolis Zoo (*N* = 4 males, 2 females), and secretion samples from ‘unknown’ donors of the opposite sex at the DLC (*N* = 16 males, 20 females) and Indianapolis Zoo (*N* = 1 male, 9 females).

Following previously established protocols [16, 40, 105], we conducted bioassays during the breeding season of 2011 and 2012. Because the subjects lived socially in multi-chambered enclosures, focal animals were temporarily isolated for bioassays, a process to which they had been accustomed. We encouraged the focal animal into a room by itself, then closed the pass-through between this room and the rest of the enclosure. We allowed samples to thaw at ambient temperature, then secured a row of three fresh wooden dowels to the fence of the animal’s test enclosure (at a 45° angle to the ground and separated by 20 cm). Using pre-cleaned forceps, we removed the thawed swab and rubbed the donor’s secretions (for ~ 10–15 s) on a predetermined dowel. The center dowel served as an unscented control, whereas a ~ 2 cm area (at lemur nose level) of the outer dowels was rubbed with a scented swab. The outer dowels thus carried scent, each from different donors, simulating two naturally placed scent marks.

Each recipient underwent 1–3 trials per day over 4–6 days, with each trial lasting 10 min, ultimately participating in 8–12 trials in total. We presented the secretions to each recipient in a randomized order. We also maximized the number of donor dyads whose secretions could be presented across recipients, while minimizing the number of times we presented secretions from each donor to any recipient (average ± S.D. exposures = 1.85 ± 1.05, range = 0–6). Recipient-donor pairs were chosen blindly with respect to donor location or MHC-DRB genotype, and not all donors were presented to all recipients, owing to logistical constraints described in the Additional file 1. Upon completion of the day’s trials, the recipient was reunited with its group.

The bioassays were videotaped, and the videos were scored using an established ethogram [105], by three observers who were blind to the MHC genotypes of the bioassay donors and recipients. Prior to scoring experimental trials, we calculated inter-observer reliability [72] from five ‘practice’ trials. Differences in the labeling of an event or in the chronology or timing (> 1 s) were considered disagreements [105] and scoring of videos did not commence until inter-observer reliability scores exceeded 90%. The main behavior recorded included investigation (e.g. sniffing and licking) and scent marking behavior (e.g. genital marking and, for males only, shoulder rubbing and wrist marking; Additional file 1: Table S2, adapted from [105]). Sniffing allows the intake of volatile information via the nasal epithelium, whereas licking is generally thought to transport non-volatile chemicals directly from the scent source to the vomeronasal organ [29]. We also recorded where investigatory or scent-marking behavior occurred relative to each scent ‘mark’ (i.e., whether the behavior was directed at the mark itself, adjacent to the mark, but on the dowel, or within 15 cm of the dowel). The placement of countermarks could have particular significance: Overmarking or placing one’s mark directly on top of the original mark might mask the original mark, whereas adjacent-marking or placing one’s mark near the original mark leaves the original mark intact [29].

### Statistical analyses

#### General analytical procedures

To examine the relationships between MHC-DRB genotype and olfactory ornamentation, as well as the ability of ring-tailed lemurs to discriminate MHC genotype via genital secretion, we analyzed the data in a series of generalized linear mixed models (GLMMs), using the package ‘glmmADMB’ (Version 0.7.7) in RStudio (Version 3.2.2; [97]). MHC diversity can be measured in various ways, including as the number of different MHC-DRB nucleotide sequences (or alleles) and as the number of MHC-DRB supertypes, which putatively predicts functional antigen binding capabilities [27, 103]. Because the number of alleles and the number of supertypes are positively correlated, we evaluated these explanatory genetic variables with independent GLMMs and used Akaike information criteria (AIC) values to determine the best-fit model [128]. We considered the model with the lowest AIC value (ΔAIC ≥2; [15]) to be the best and report only those models in the main text, although the AIC values for all models are reported in the Additional file 1: Tables S3, S4, S5 and S7). Because the sexes are dimorphic in their glands [106], and thus, their marking behavior, we treated the sexes separately in the analyses of both chemical diversity and behavioral discrimination.

To examine if the similarity in MHC between individuals was reflected in their chemical similarity, we used partial Mantel tests to compare the number of un-shared or unique MHC-DRB alleles and supertypes to the relative Euclidean distance matrices between male-male (MM), female-female (FF), and male-female (MF) dyads. For consistency, we report results for MHC-DRB supertypes in the main text and results for MHC-DRB alleles in the Additional file 1: Table S6.

#### Analyses of MHC-DRB diversity and chemical complexity in individual males and females

To examine the relationship between MHC-DRB diversity and chemical complexity of the labial or scrotal secretions, each chemical diversity index was evaluated in a separate series of GLMMs using Gaussian distribution with donor identity as a random variable. Explanatory variables included season (i.e., breeding and nonbreeding) and either the number of MHC-DRB alleles (MHC_allele_) or the number of MHC-DRB supertypes (MHC_supertype_) possessed by an individual donor. Because of skew in the frequency of specific MHC-DRB supertypes (i.e., seven supertypes were found in fewer than five individuals, whereas one supertype was found in more than 85% of individuals), we were unable to examine if possession of a specific supertype could be signaled via the chemical complexity of genital secretions. For both sexes, we also analyzed genetic diversity in relation to the chemical diversity of FAs and FAEs [10]. Where patterns for chemical diversity of all the compounds in a genital secretion reflect the patterns for chemical diversity of FAs and FAEs, we report only the results for the chemical diversity of all compounds.

We were unable to control for neutral heterozygosity estimated via microsatellites (see [10, 16] for microsatellite methods) because microsatellite data were unavailable for > 20% of our subjects. Nevertheless, we assessed the correlation between microsatellite heterozygosity and MHC-DRB diversity to determine if microsatellite diversity might explain the pattern of results. Using a linear regression on the subset of subjects for which both genetic measures of diversity were available (*N* = 36), we found no correlation between microsatellite heterozygosity and the number of MHC-DRB alleles within an individual (slope = 1.056, F = 1.51, *P* = 0.227), and no correlation between microsatellite heterozygosity and the number of MHC-DRB supertypes (slope = − 0.8812, F = 1.461, *P* = 0.235; see also [42]). As we previously had detected no relationships between individual chemical diversity and adult age, the month of collection within season, or DLC housing condition [16], we did not include these co-variables in our analyses of the relationship between MHC-DRB diversity and chemical diversity.

The captive ring-tailed lemur population lacks genetic diversity compared to wild populations [42]. Notably, our captive subjects included only two (*N* = 1 male, 1 female) relatively MHC-diverse individual. Because these individuals were representative of the average MHC-DRB diversity present in wild populations [42], we did not consider them to be outliers; nevertheless, to verify that they were not driving the association between MHC-DRB diversity and chemical diversity, we re-ran the final GLMMs after removing the most diverse individuals the datasets (*N* = 1; Additional file 1: Tables S3, S4 and S5). Because all three measures of chemical diversity (i.e., richness, Shannon index, and Simpson index) showed the same patterns when compared to either measure of genetic diversity (i.e., allele or supertype number), we report only the GLMM with the lowest AIC values [15] in the main text.

#### Analysis of MHC relatedness and chemical similarity between all possible dyads

We used partial Mantel tests to investigate if the chemical similarity between dyads reflected the similarity in their MHC genotypes. First, we calculated matrices of genetic distances using the number of different MHC alleles and supertypes between each dyad. We then estimated the chemical distances between pairs of individuals, by analyzing all of the chemical compounds identified in secretion profiles of either sex (*n* = 203 compounds for males: [16]; *n* = 338 for females: [10]), or only those compounds shared by both sexes (*n* = 170 compounds: [9]). We calculated relative Euclidean distance matrices for same-sex (MM or FF) and mixed-sex (MF) dyads, respectively, using PC-ORD (version 7.0, [76]), and following published protocols [9, 16]. We calculated matrices separately as follows: breeding season (for *N* = 22 males, there were 231 MM dyads; for *N* = 17 females, there were 136 FF dyads; and for *N* = 39 males and females, there were 374 MF dyads); nonbreeding season (for *N* = 20 males, there were 190 MM dyads; for *N* = 18 females, there were 153 FF dyads; and for *N* = 38 males and females, there were 360 MF dyads). Because MM and FF matrices were square, we assessed linear relationships between chemical and MHC distances using partial Mantel tests in FSTAT (version 2.9.3.2, with 10,000 randomizations; [38]). As in previous studies [10, 16], we controlled for potentially confounding covariates, including the subject’s age, social housing condition, and the month of secretion sample collection. For the MF comparisons, we first generated full matrices using all possible MM, FF, and MF pairs (breeding season: *n* = 704 dyads; nonbreeding season: *n* = 741 dyads). We then extracted chemical, genetic, and covariate information for MF dyads only. Unlike MM and FF matrices, the MF matrix was not square. Therefore, we assessed relationships with 10,000 Spearman’s correlation permutation tests using the JMUOUTLIER package in R (Version 1.3; [35]), as in the study by Slade et al. [112].

Lastly, to confirm that our results were not being driven by the overall genetic similarity between dyads, rather than by allelic sharing at the MHC-DRB loci, we assessed the correlation between MHC similarity within dyads (i.e., the number of unique or unshared MHC-DRB alleles and supertypes between two individuals) with dyad relatedness, as measured by the Queller and Goodnight index (IDQG calculated in [10]). Although dyad relatedness was significantly and negatively correlated with MHC dissimilarity for both the number of MHC-DRB alleles (*n* = 629 dyads, slope = − 0.71, T-value = − 4.21, *P* = 0.000029) and the number of MHC-DRB supertypes (*n* = 629 dyads, slope = − 0.67, T-value = − 4.23, *P* = 0.000027), the negative relationships explained less than 3% of the variance in either correlation (*R*^*2*^ = 0.026 and *R*^*2*^ = 0.026, respectively). Because the partial Mantel tests for both the unique MHC alleles and the unique supertypes showed similar patterns, we report supertype results in the main text and allelic results in the Additional file 1.

#### Behavioral analyses of mixed-sex, recipient-donor combinations

We explored the relationship between the recipients’ behavioral responses to donor secretions and measures of absolute and relative MHC-DRB diversity between the mixed-sex, recipient-donor dyads using a separate series of GLMMs for each behavioral response, with a negative binomial distribution and log link function. In each GLMM, we controlled for the random variables of trial number on a given day (i.e., 1–3), the number of times that a recipient had been presented with the secretion from a given donor over the course of the study (i.e., 1–6), as well as the secretion donor ID nested under secretion recipient.

To test for odorant discrimination, we used two measures of dissimilarity and sequence divergence between each recipient-donor dyad, the number of unique alleles and the number of unique supertypes between dyads (Additional file 1: Table S8; [51, 104, 108]). We also used the donor’s number of MHC alleles to examine if conspecifics that had the greatest MHC diversity were distinguished by their scent alone, regardless of their genetic dissimilarity (Additional file 1: Table S8). Lastly, we examined non-linear relationships between MHC diversity and dissimilarity between dyads by including the quadratic forms of all genetic explanatory variables in our GLMMs. Quadratic terms were retained only if the AIC value was better than the GLMM that included only linear terms (Additional file 1: Table S7).

Although we recorded both the frequency and duration of all behavior, we analyzed only frequencies for events and durations for states [4, 72]. In our analyses, we excluded all recipient behavior that occurred in < 5% of trials and any behavior that was not directed significantly more toward the test dowels over the control dowel, as determined via Wilcoxon signed-rank tests [18]. Ultimately, for male recipients, we analyzed the duration of time spent in proximity to the dowels and sniffing and licking the mark and surrounding areas, as well as the frequency of shoulder rubs, and wrist marking the area adjacent to the mark. For females, we analyzed the duration of time spent sniffing and licking the mark and the adjacent area. For each behavioral response, we report the genetic explanatory variable (e.g. unshared MHC-DRB alleles, unshared MHC-DRB supertypes, or the number of donor supertypes) with the lowest AIC value. The AIC values for other models are reported in the Additional file 1: Table S7. We also verified that the behavioral responses to odorants were comparable for samples collected at different facilities (i.e., DLC or Indianapolis Zoo) and across trials, regardless of trial order (Additional file 1).

## Additional file

Additional file 1: Supplementary methods and results. (DOCX 45 kb)

## Data Availability

Analyses reported in this article can be reproduced using the following datasets (i.e., CSV files of MHC, chemical, and behavioral data from bioassays) that have been deposited into Dryad at DOI: 10.5061/dryad.cb010bf.

## Abbreviations

DLC: Duke Lemur Center
FAEs: Fatty acid esters
FAs: Fatty acids
FF dyad: Female-female dyad
GCMS: Gas chromatography mass spectrometry
GLMM: General linear mixed model
MF dyad: Male-female dyad
MHC: Major Histocompatibility Complex
MHC-DRB: Major Histocompatibility Complex DRB gene
MM dyad: Male-male dyad
