# Supplementary material for: Genetic variation at MHC class II loci influences both olfactory signals and scent discrimination in ring-tailed lemurs
Source: BMC Evol Biol. 2019 Aug 22;19:171. doi: 10.1186/s12862-019-1486-0 (PMC6704550; doi:10.1186/s12862-019-1486-0)
Supplement: Supplementary file 1 — Supplementary methods and results. (DOCX 45 kb) [file 12862_2019_1486_MOESM1_ESM.docx]

**SUPPLEMENTAL METHODS**

*Subject considerations for bioassays*

Because prior interactions with a conspecific can affect the behavioral response to their scent, we only presented bioassay recipients with odorants collected from donors that were ‘unknown’ to the recipient. We defined unknown as never having lived concurrently in the same social group as the recipient, either because the donor resided at a different facility or resided at the Duke Lemur Center (DLC), but in a different social group. At the DLC, social groups of the same species are kept physically separate at all times to prevent injury from antagonistic interactions and to prevent sub-optimal reproductive partnerships. We verified that no animal that participated in this study had been transferred between the three captive locations. Additionally, donors were only considered unknown if their odorants had never been presented to the recipient in previous behavioral bioassays conducted for any previous studies (e.g. Scordato & Drea 2007, Charpentier et al. 2010, Crawford et al. 2011, Greene et al. 2016).

*Sampling logistics*

Not all of the subjects could serve as secretion donors for chemical analyses or bioassay presentation, nor could all of the subjects participate as bioassay recipients. To achieve appropriate sample sizes while working with a captive, endangered species often requires years of sample collection and observation, which presents various logistical challenges (for more details, see Drea et al. 2013). Specific to our work here, secretion sampling was restricted because of several factors. These included facility practices that limit the frequency of animal captures, researcher efforts to avoid confounds from hormonal contraception (Crawford et al. 2011) or pregnancy (Crawford & Drea 2015), and constraints imposed by animal mortality or transfer between facilities. As detailed in the main text, participation as a bioassay recipient or donor may also have been precluded because of participation in previous studies, leading to either a lack of ‘unknown’ donor odorants to present to a recipient or a lack of recipients that had never been presented with that individual’s secretions. For this study, we overcame these challenges whenever possible through long-term study and through the addition of animals from other facilities.

*Additional behavioral analyses of mixed-sex, recipient-donor combinations*

To verify that the responses to odorants collected at different facilities (the DLC or Indianapolis Zoo) from animals under different capture conditions (e.g. awake or anesthetized) did not influence our findings, we compared the total number of seconds that recipients spent responding to each type of odorant. The responses to odorants collected from anesthetized animals (males: *N* = 49, Mean ± SD = 38.0 ± 44.0; females: *N* = 6, Mean ± SD = 2.5 ± 2.43) were within the range of responses to odorants collected from awake animals (males: *N* = 260, Mean ± SD = 50.4 ± 38.0; females: *N* = 167, Mean ± SD = 7.9 ± 10.0).

We also verified that recipient subjects did not become ‘habituated’ to the bioassay task, such that participation in the trials did not change from the first bioassay to the last bioassay, by comparing the total duration of behavior directed towards dowels by bioassay number. While overall participation in the bioassay decreased for both sexes, this decrease explained little of the variation in time of participation across trials (Males: slope = -3.77, t = -4.64, *P* < 0.001, R^2^ = 0.06; Females: slope = -0.85, t = -3.46, *P* < 0.001, R^2^ = 0.06).

**Table S1.** Summary of individual and population-level MHC-DRB genotype information, with columns for the average number of MHC-DRB alleles and supertypes detected per individual, the total number of alleles detected per population, the total number of supertypes detected per population, and the number of alleles and supertypes unique to that population, respectively.

|  | **Per individual** | | **Per population** | | | |
| --- | --- | --- | --- | --- | --- | --- |
|  | Average allele number | Average supertype number | Total alleles | Total supertypes | Unique alleles | Unique supertypes |
| Duke Lemur Center (*N* = 48) | 2.1 | 1.7 | 16 | 10 | 9 | 4 |
| Indianapolis Zoo (*N* = 12) | 2.5 | 1.9 | 11 | 8 | 2 | 0 |
| Cincinnati Zoo (*N* = 2) | 3.5 | 3.5 | 4 | 4 | 0 | 0 |

**Table S2**. Behavioral ethogram of analyzed behaviors, modified from Scordato & Drea 2007.

| Behavior | Definition |
| --- | --- |
| **Proximity** | Subject is within 15 cm of a dowel, as judged by the position of the subject’s nose. Scored as the duration of time spent in proximity, terminated when subject moves head or whole body away from the dowel. |
| **Sniff** | Subject places nose at least 1 cm away from scent mark. Scored as a duration in which the sniffing bout ends after subject moves nose more than 1 cm away for more than 1 second. A distinction is made between ‘sniff scent’ (i.e. nose directly over the area of odorant deposition), and ‘sniff substrate’ (i.e. sniffing behavior directed elsewhere on the dowel). Sniffing reflects olfactory investigation of the volatile portion of the odorant. |
| **Lick** | Subject touches scent mark with tongue. Scored both for the duration of the licking bout, and frequency of individual licks, with a bout ending after the subject ceases licking for more than 1 second. As with sniffing, a distinction is made between ‘lick scent’ and ‘lick substrate’. Licking indicates gustatory investigation of the nonvolatile fraction of the scent mark. |
| **Shoulder Rub** | Male subject brings his wrist up to his shoulder and rubs his antebrachial organ against his brachial organ, presumably mixing the secretions from both glands. Scored for both frequency of rubs and duration of each shoulder rub. |
| **Wrist Mark** | Male subject draws antebrachial gland across substrate. Scored for both frequency of marks and duration of each mark. As with genital marking, a distinction is made between ‘countermarking’ and ‘adjacent marking’. |

**Table S3.** Summary of the models used to determine the best explanatory genetic variable (i.e., allelic diversity, MHC_allele_, or supertype diversity, MHC_supertype_) in the analysis of MHC diversity and odorant complexity in (3A) male and (3B) female ring-tailed lemurs across seasons. For each model, the individual donor was included as a random variable and all models were run with a Gaussian data distribution and identity link function. Significant relationships (*P* ≤ 0.05) are shown in bold and trending relationships (*P* ≤ 0.10) are shown in italics. In the main text, we report the GLMM for which ΔAIC ≥ 2 (Burnham & Anderson 2002), indicated by (‡). When two GLMMs had ‘equivalent’ AICs values (ΔAIC ≥ 2), we report the model that was most consistent with all of the results. In each table, GLMM #7 is a variation of the reported GLMM, with the most MHC-DRB diverse individual of that sex excluded. AIC values are not reported for these models, as the datasets between models 7 and models 1-6 are not equal.

**Table S3A.** Males

| **Model** | **Best-fit explanatory variables** | **AIC** | **ΔAIC** | **Z value** | ***P* value** |
| --- | --- | --- | --- | --- | --- |
| 1) Richness-overall | Season  MHC_supertype_  Season x MHC_supertype_ | 342.1 | 635.6 | **2.37**  **2.31**  -0.71 | **0.018**  **0.021**  0.475 |
| 2) Richness-overall | Season  MHC_allele_  Season x MHC_allele_ | 346.0 | 639.5 | *1.67*  1.09  -0.31 | *0.096*  0.277  0.755 |
| 3) Shannon Index-overall | Season  MHC_supertype_  Season x MHC_supertype_ | -4.7 | 288.8 | **2.43**  **2.52**  -1.45 | **0.015**  **0.012**  0.146 |
| 4) Shannon Index-overall | Season  MHC_allele_  Season x MHC_allele_ | 0.6 | 294.1 | 0.69  0.68  0.12 | 0.490  0.500  0.910 |
| 5) Simpson Index-overall ‡ | Season  MHC_supertype_  Season x MHC_supertype_ | -293.5 | 0 | 1.64  **2.17**  -0.89 | 0.100  **0.030**  0.370 |
| 6) Simpson Index-overall | Season  MHC_allele_  Season x MHC_allele_ | -289.7 | 3.8 | 0.51  0.47  0.14 | 0.610  0.470  0.890 |
| 7) Simpson Index overall - with most diverse male dropped from dataset | Season  MHC_supertype_  Season x MHC_supertype_ | NA | NA | *1.86*  **2.13**  -1.22 | *0.062*  **0.033**  0.221 |

**Table S3B.** Females

| **Measure of chemical diversity** | **Best-fit explanatory variables** | **AIC** | **ΔAIC** | **Z value** | ***P* value** |
| --- | --- | --- | --- | --- | --- |
| 1) Richness-overall | Season  MHC_supertype_  Season x MHC_supertype_ | 328.7 | 612.2 | -0.53  -1.02  -0.92 | 0.600  0.310  0.360 |
| 2) Richness-overall | Season  MHC_allele_  Season x MHC_allele_ | 330.0 | 613.5 | -0.71  -0.93  -0.55 | 0.480  0.350  0.580 |
| 3) Shannon Index-overall | Season  MHC_supertype_  Season x MHC_supertype_ | -21.9 | 261.6 | 0.77  -0.34  *-1.69* | 0.442  0.733  *0.091* |
| 4) Shannon Index-overall | Season  MHC_allele_  Season x MHC_allele_ | -23.8 | 259.7 | 0.94  -0.49  *-1.79* | 0.349  0.628  *0.074* |
| 5) Simpson Index-overall ‡ | Season  MHC_supertype_  Season x MHC_supertype_ | -282.7 | 0.8 | 0.86  0.24  -0.98 | 0.390  0.810  0.330 |
| 6) Simpson Index-overall | Season  MHC_allele_  Season x MHC_allele_ | -283.5 | 0 | 0.89  -0.09  -0.99 | 0.380  0.930  0.320 |
| 7) Simpson Index overall - with most diverse female dropped from dataset | Season  MHC_supertype_  Season x MHC_supertype_ | NA | NA | 0.08  -0.16  0.01 | 0.930  0.880  0.990 |

**Table S4.** Summary of the GLMMs explored for the best explanatory genetic variable (i.e., allelic diversity, MHC_allele_, or supertype diversity, MHC_supertype_) between measures of the chemical diversity of (4A) fatty acids, or FAs, or (4B) fatty acid esters, or FAEs, and MHC diversity in male ring-tailed lemurs across seasons, with significant relationships (*P* ≤ 0.05) shown in bold and trending relationships (*P* ≤ 0.10) shown in italics. For each model, the individual was included as a random variable and all models were run with a Gaussian data distribution and identity link function. In each table, ‡ indicates the model reported in the main text. The last row in each table shows the reported GLMM with the most MHC-DRB diverse male excluded. AIC values are not reported for these models, as the datasets between models 7 and models 1-6 are not equal.

**Table S4A.** Fatty acids in males

| **Measure of chemical diversity** | **Best-fit explanatory variables** | **AIC** | **ΔAIC** | **Z value** | ***P* value** |
| --- | --- | --- | --- | --- | --- |
| 1) Richness - FAs | Season  MHC_supertype_  Season x MHC_supertype_ | 248.7 | 339.5 | *1.69*  0.56  -0.15 | *0.091*  0.576  0.878 |
| 2) Richness - FAs | Season  MHC_allele_  Season x MHC_allele_ | 248.0 | 338.8 | 1.51  -0.66  -0.31 | 0.130  0.510  0.760 |
| 3) Shannon - FAs | Season  MHC_supertype_  Season x MHC_supertype_ | 30.6 | 121.2 | **1.97**  **2.12**  -0.54 | **0.049**  **0.034**  0.589 |
| 4) Shannon - FAs | Season  MHC_allele_  Season x MHC_allele_ | 31.6 | 124.1 | **2.65**  *1.92*  -1.53 | **0.009**  *0.055*  0.126 |
| 5) Simpson – FAs | Season  MHC_supertype_  Season x MHC_supertype_ | -89.1 | 1.7 | *1.83*  **2.11**  -0.78 | *0.068*  **0.035**  0.438 |
| 6) Simpson - FAs ‡ | Season  MHC_allele_  Season x MHC_allele_ | -90.8 | 0.0 | **2.42**  **2.55**  -1.52 | **0.016**  **0.011**  0.128 |
| 7) Simpson – FAs with the most diverse male dropped from the dataset | Season  MHC_allele_  Season x MHC_allele_ | NA | NA | **2.34**  **2.26**  -1.48 | **0.019**  **0.024**  0.138 |

**Table S4B.** Fatty acid esters in males

| **Measure of chemical diversity** | **Best-fit explanatory variables** | **AIC** | **ΔAIC** | **Z value** | ***P* value** |
| --- | --- | --- | --- | --- | --- |
| 1) Richness - FAEs | Season  MHC_supertype_  Season x MHC_supertype_ | 260.4 | 515.5 | 0.55  1.48  0.49 | 0.580  0.140  0.620 |
| 2) Richness - FAEs | Season  MHC_allele_  Season x MHC_allele_ | 262.7 | 517.8 | 0.67  1.00  0.16 | 0.500  0.320  0.870 |
| 3) Shannon Index - FAEs | Season  MHC_supertype_  Season x MHC_supertype_ | -9.8 | 245.3 | *1.93*  **2.26**  -1.17 | *0.054*  **0.024**  0.241 |
| 4) Shannon - FAEs | Season  MHC_allele_  Season x MHC_allele_ | -5.8 | 249.3 | 0.71  0.78  -0.08 | 0.480  0.440  0.940 |
| 5) Simpson Index - FAEs ‡ | Season  MHC_supertype_  Season x MHC_supertype_ | -255.1 | 0.0 | *1.77*  **2.18**  -1.03 | *0.076*  **0.029**  0.305 |
| 6) Simpson – FAEs | Season  MHC_allele_  Season x MHC_allele_ | -251.6 | 3.5 | 0.74  0.90  -0.11 | 0.460  0.370  0.910 |
| 7) Simpson – FAEs with the most diverse male dropped from the dataset | Season  MHC_allele_  Season x MHC_allele_ | NA | NA | **2.13**  **2.35**  -1.49 | **0.033**  **0.019**  0.137 |

**Table S5.** Summary of the GLMMs explored for the best explanatory genetic variable (i.e., allelic diversity, MHC_allele_, or supertype diversity, MHC_supertype_) between measures of chemical diversity of (5A) fatty acids, or FAs, or (5B) fatty acid esters, or FAEs, and MHC diversity in female ring-tailed lemurs across seasons, with significant relationships (*P* ≤ 0.05) shown in bold and trending relationships (*P* ≤ 0.10) shown in italics. For each model, individual was included as a random variable and all models were run with a gaussian data distribution and identity link function. In each table, ‡ indicates the model reported in the main text. The last row in each table shows the reported GLMM with the most MHC-DRB diverse female excluded. AIC values are not reported for these models, as the datasets between models 7 and models 1-6 are not equal.

**Table S5A.** Fatty acids in females

| **Measure of chemical diversity** | **Best-fit explanatory variables** | **AIC** | **ΔAIC** | **Z value** | ***P* value** |
| --- | --- | --- | --- | --- | --- |
| 1) Richness - FAs | Season  MHC_supertype_  Season x MHC_supertype_ | 230.9 | 316.0 | 0.37  0.27  -1.56 | 0.710  0.790  0.120 |
| 2) Richness - FAs | Season  MHC_allele_  Season x MHC_allele_ | 233.8 | 319.0 | -0.61  0.08  -0.36 | 0.550  0.940  0.720 |
| 3) Shannon - FAs | Season  MHC_supertype_  Season x MHC_supertype_ | 30.7 | 115.9 | 1.62  -0.20  **-3.09** | 0.104  0.838  **0.002** |
| 4) Shannon - FAs | Season  MHC_allele_  Season x MHC_allele_ | 41.5 | 126.7 | -0.42  -0.80  -0.54 | 0.680  0.420  0.590 |
| 5) Simpson – FAs ‡ | Season  MHC_supertype_  Season x MHC_supertype_ | -85.2 | 0 | **2.24**  -0.38  **-3.75** | **0.025**  0.703  **0.001** |
| 6) Simpson - FAs | Season  MHC_allele_  Season x MHC_allele_ | -70.4 | 14.8 | 0.06  -0.75  -0.98 | 0.950  0.450  0.330 |
| 7) Simpson – FAs with most diverse female dropped from dataset | Season  MHC_supertype_  Season x MHC_supertype_ | NA | NA | 1.37  0.52  **-2.17** | 0.170  0.600  **0.030** |

**Table S5B.** Fatty acid esters in females

| **Measure of chemical diversity** | **Best-fit explanatory variables** | **AIC** | **ΔAIC** | **Z value** | ***P* value** |
| --- | --- | --- | --- | --- | --- |
| 1) Richness - FAEs | Season  MHC_supertype_  Season x MHC_supertype_ | 236.5 | 516.1 | -1.00  **-1.99**  -0.59 | 0.316  **0.046**  0.557 |
| 2) Richness - FAEs | Season  *MHC_allele_*  Season x MHC_allele_ | 234.3 | 513.9 | 0.01  *-1.69*  -0.45 | 0.994  *0.092*  0.656 |
| 3) Shannon Index - FAEs | Season  MHC_supertype_  Season x MHC_supertype_ | -36.7 | 242.9 | 0.08  -0.65  -1.16 | 0.940  0.520  0.250 |
| 4) Shannon - FAEs | Season  MHC_allele_  Season x MHC_allele_ | -45.3 | 234.3 | 1.43  -0.42  **-2.63** | 0.152  0.678  **0.009** |
| 5) Simpson Index - FAEs | Season  MHC_supertype_  Season x MHC_supertype_ | -275 | 4.6 | 0.15  -0.34  -0.76 | 0.880  0.740  0.450 |
| 6) Simpson – FAEs ‡ | Season  MHC_allele_  Season x MHC_allele_ | -279.6 | 0 | 1.02  -0.34  -1.64 | 0.310  0.740  0.100 |
| 7) Simpson – FAEs with most diverse female dropped from dataset | Season  MHC_allele_  Season x MHC_allele_ | NA | NA | 0.12  -0.47  -0.35 | 0.900  0.640  0.730 |

**Table S6**. Partial Mantel tests for same-sex (MM and FF) dyads, showing seasonal relationships between relative Euclidean chemical distance and MHC-based genetic distance (number of unique MHC alleles) in ring-tailed lemurs. Chemical distance is based on 203 and 338 compounds for MM and FF dyads, respectively. Tests include three socio-demographic and environmental variables as covariates. Sums of squares (SS) and partial Mantel correlation coefficients (*r*) with significant *P* values (*P* ≤ 0.05) are shown in bold type, whereas trending values (*P* ≤ 0.10) are shown in italics.

| Dyad type | Variable | Number of unique MHC alleles | | | | | |
| --- | --- | --- | --- | --- | --- | --- | --- |
|  |  | Breeding season | | | Nonbreeding season | | |
|  |  | SS | *r* | *P* | SS | *r* | *P* |
| MM dyads | MHC | **1.502** | **0.329** | **<0.001** | 0.038 | -0.076 | 0.299 |
|  | Age | *0.194* | *0.118* | *0.072* | **0.139** | **0.145** | **0.004** |
|  | Housing | 0.159 | 0.107 | 0.104 | 0.008 | -0.035 | 0.634 |
|  | Month of collection | 0.030 | -0.047 | 0.477 | *0.099* | *0.123* | *0.089* |
| FF dyads | MHC | **0.244** | **0.247** | **0.003** | 0.015 | -0.052 | 0.516 |
|  | Age | <0.001 | 0.009 | 0.908 | <0.001 | -0.002 | 0.978 |
|  | Housing | 0.055 | 0.118 | 0.179 | 0.028 | 0.073 | 0.386 |
|  | Month of collection | *0.103* | *0.161* | *0.061* | **1.434** | **0.517** | **<0.001** |

**Table S7**. Summary of the GLMMS explored for the best explanatory genetic variable of (A) male and (B) female behavioral responses during behavioral bioassays. The allele difference and supertype difference reflect relative dissimilarity between the recipient-donor dyad, whereas the donor allele number represents the absolute MHC-diversity of the donor. All GLMMs included the random variables: daily trial number, recipient experience with a particular donor’s secretions, and donor identity nested within recipient identity. NAs indicate the model failed to converge and ΔAIC could not be calculated. **‡** indicates the final model reported.

**Table S7A**. Males

| **Model Formula** | **AIC** | **ΔAIC** | |
| --- | --- | --- | --- |
| Lick Mark Duration ~ Allele Difference  Lick Mark Duration ~ Allele Difference + Allele Difference^2^  Lick Mark Duration ~ Supertype Difference  Lick Mark Duration ~ Supertype Difference + Supertype Difference^2^  Lick Mark Duration ~ Donor Allele Number  Lick Mark Duration ~ Donor Allele Number + Donor Allele Number^2^ ‡ | 516.9  518.0  509.7  NA  511.7  496.1 | 20.8  21.9  13.6  NA  15.6  0.0 |  |
| Proximity Duration ~ Allele Difference  Proximity Duration ~ Allele Difference + Allele Difference^2^  Proximity Duration ~ Supertype Difference ‡  Proximity Duration ~ Supertype Difference + Supertype Difference^2^  Proximity Duration ~ Donor Allele Number  Proximity Duration ~ Donor Allele Number + Donor Allele Number^2^ | 2440.1  2442.0  2439.9  2441.8  2439.9  2440.9 | 0.2  2.1  0.0  1.9  0.0  1.0 |  |
| Shoulder Rub Frequency ~ Allele Difference  Shoulder Rub Frequency ~ Allele Difference + Allele Difference^2^  Shoulder Rub Frequency ~ Supertype Difference ‡  Shoulder Rub Frequency ~ Supertype Difference + Supertype Difference^2^  Shoulder Rub Frequency ~ Donor Allele Number  Shoulder Rub Frequency ~ Donor Allele Number + Donor Allele Number^2^ | 843.4  845.1  841.9  843.7  845.9  846.3 | 1.5  3.2  0.0  1.8  4.0  4.4 |  |
| Smell Mark Duration ~ Allele Difference  Smell Mark Duration ~ Allele Difference + Allele Difference^2^  Smell Mark Duration ~ Supertype Difference  Smell Mark Duration ~ Supertype Difference  Smell Mark Duration ~ Donor Allele Number  Smell Mark Duration ~ Donor Allele Number + Donor Allele Number^2^ ‡ | 2193.1  2194.8  2190.3  2192.0  2191.8  2189.2 | 3.9  5.6  1.1  2.8  2.6  0.0 |  |
| Smell Dowel Duration ~ Allele Difference  Smell Dowel Duration ~ Allele Difference + Allele Difference^2^  Smell Dowel Duration ~ Supertype Difference  Smell Dowel Duration ~ Supertype Difference + Supertype Difference^2^ ‡  Smell Dowel Duration ~ Donor Allele Number  Smell Dowel Duration ~ Donor Allele Number + Donor Allele Number^2^ | 1658.3  1659.9  1659.7  1657.8  1663.3  1664.8 | 0.5  2.1  1.9  0.0  5.5  7.0 |  |
| Wrist Mark Frequency ~ Allele Difference  Wrist Mark Frequency ~ Allele Difference + Allele Difference^2^  Wrist Mark Frequency ~ Supertype Difference  Wrist Mark Frequency ~ Supertype Difference + Supertype Difference^2^  Wrist Mark Frequency ~ Donor Allele Number ‡  Wrist Mark Frequency ~ Donor Allele Number + Donor Allele Number^2^ | NA  NA  1102.2  1103.7  1101.0  1102.3 | NA  NA  1.2  2.7  0.0  1.3 |  |

**Table S7B.** Females

| **Behavioral Variable** | **AIC** | **ΔAIC** |
| --- | --- | --- |
| Lick Mark Duration ~ Allele Difference  Lick Mark Duration ~ Allele Difference + Allele Difference^2^  Lick Mark Duration ~ Supertype Difference  Lick Mark Duration ~ Supertype Difference + Supertype Difference^2^  Lick Mark Duration ~ Donor Allele Number ‡  Lick Mark Duration ~ Donor Allele Number + Donor Allele Number^2^ | 251.4  252.8  251.9  253.8  250.4  NA | 1.0  2.4  1.5  3.4  0.0  NA |
| Smell Mark Duration ~ Allele Difference  Smell Mark Duration ~ Allele Difference + Allele Difference^2^  Smell Mark Duration ~ Supertype Difference  Smell Mark Duration ~ Supertype Difference + Supertype Difference^2^  Smell Mark Duration ~ Donor Allele Number ‡  Smell Mark Duration ~ Donor Allele Number + Donor Allele Number^2^ | 916.9  918.8  917.1  918.2  916.4  918.4 | 0.5  2.4  0.7  1.7  0.0  2.0 |
| Smell Dowel Duration ~ Allele Difference  Smell Dowel Duration ~ Allele Difference + Allele Difference^2^  Smell Dowel Duration ~ Supertype Difference ‡  Smell Dowel Duration ~ Supertype Difference + Supertype Difference^2^  Smell Dowel Duration ~ Donor Allele Number  Smell Dowel Duration ~ Donor Allele Number + Donor Allele Number^2^ | 620.2  621.1  619.4  620.7  NA  NA | 0.8  1.7  0.0  1.3  NA  NA |

**Table S8.** Explanatory variables used in the analysis of recipient responses to conspecific odorants.

| **Variable** | **Definition** |
| --- | --- |
| **MHC_supertype diff_** | The number of MHC-DRB supertypes that were different between a recipient-donor dyad |
| **MHC_allele diff_** | The number of MHC-DRB alleles that were different between a recipient-donor dyad |
| **MHC_donor_** | The number of MHC-DRB alleles that were present in the donor |

**REFERENCES**

Burnham KP & Anderson DR. 2002. Model Selection and Multimodel Inference: A Practical Information-Theoretic Approach. New York, New York. Springer.

Charpentier MJE, Crawford JC, Boulet M, & Drea CM. 2010. Message 'scent': lemurs detect the genetic relatedness and quality of conspecifics via olfactory cues. *Animal Behaviour* 80: 101-108.

Crawford JC, Boulet M, & Drea CM. 2011. Smelling wrong: hormonal contraception in lemurs alters critical female odour cues. *Proceedings of the Royal Society of London, B Biological Sciences* 278 (1702): 122-130.

Crawford J & Drea CM. 2015. Baby on board: olfactory cues indicate pregnancy and fetal sex in a non-human primate. *Biology Letters* 11(2): 20140831.

Drea CM, Boulet M, delBarco-Trillo J, Greene LK, Sacha CR, Goodwin, TE & Dubay GR. 2013. The “secret” in secretions: Methodological considerations in deciphering primate olfactory communication. *American Journal of Primatology* 75 (7): 621-642.

Greene LK, Grogan KE, Smyth KN, Adams CA, Klager SA, & Drea CM. 2016. Mix it and fix it: functions of composite olfactory signals in ring-tailed lemurs. *Royal Society Open Science* 3: 160076.

Scordato ES & Drea CM. 2007. Scents and sensibility: Information content of olfactory signals in the ringtailed lemur (*Lemur catta*). *Animal Behaviour* 73 (2): 301-314.
